# Protein Microarray On-Demand: A Novel Protein Microarray System

**DOI:** 10.1371/journal.pone.0003265

**Published:** 2008-09-24

**Authors:** Deb K. Chatterjee, Kalavathy Sitaraman, Cassio Baptista, James Hartley, Thomas M. Hill, David J. Munroe

**Affiliations:** 1 Protein Expression Laboratory, SAIC-Frederick, Inc., NCI-Frederick, Frederick, Maryland, United States of America; 2 Laboratory of Molecular Technology, SAIC-Frederick, Inc., NCI-Frederick, Frederick, Maryland, United States of America; 3 Advanced Technology Program, SAIC-Frederick, Inc., NCI-Frederick, Frederick, Maryland, United States of America; 4 Department of Microbiology and Immunology, School of Medicine and Health Sciences, University of North Dakota, Grand Forks, North Dakota, United States of America; Baylor College of Medicine, United States of America

## Abstract

We describe a novel, simple and low-cost protein microarray strategy wherein the microarrays are generated by printing expression ready plasmid DNAs onto slides that can be converted into protein arrays on-demand. The printed expression plasmids serve dual purposes as they not only direct the synthesis of the protein of interest; they also serve to capture the newly synthesized proteins through a high affinity DNA-protein interaction. To accomplish this we have exploited the high-affinity binding (∼3–7×10 ^−13^ M) of *E. coli* Tus protein to Ter, a 20 bp DNA sequence involved in the regulation of *E. coli* DNA replication. In our system, each protein of interest is synthesized as a Tus fusion protein and each expression construct directing the protein synthesis contains embedded Ter DNA sequence. The embedded Ter sequence functions as a capture reagent for the newly synthesized Tus fusion protein. This “all DNA” microarray can be converted to a protein microarray on-demand without need for any additional capture reagent..

## Introduction

The rapid development of genomic databases, bioinformatics tools, laboratory robotics and enabling technologies such as cDNA and oligonucleotide microarrays have provided new insights and understanding into biological and disease processes thru the global analysis of gene expression patterns. Continued development of high-throughput platforms, such as protein microarray technologies, are essential to furthering our understanding of protein function, quantitative proteomics, molecular interactions and protein profiling [1]–[3]. Unfortunately, inherent cost and technical limitations, including the required production of large libraries of purified proteins and long-term maintenance of array stability and integrity, have caused protein microarray development to lag behind that of DNA microarrays [2], [4]. Nevertheless, despite these limitations, several groups have demonstrated proof-of-concept and the potential of protein microarray technology [4]–[9].

In an effort to address these issues, Nord et al. developed an alternative platform, termed protein microbead display, wherein proteins are captured *via* antigen-antibody binding as they are synthesized [10]. This technology utilizes a biotin labeled PCR product (containing a T7 promoter and a FLAG epitope in-frame with two IgG binding domains) anchored onto microbeads through streptavidin-biotin affinity binding. Anti-FLAG antibody is then immobilized onto the same microbead. The beads are incubated in a coupled cell-free transcription-translation extract to produce the targeted protein. Newly synthesized proteins are trapped via Flag peptide (antigen)-Flag antibody interaction. More recently, Ramachandran et al. [11] applied a similar antibody mediated protein anchoring technology to a microarray format. This platform employs purified expression construct DNAs arrayed onto a microscope slide *via* biotin-avidin interaction. The encoded inserts are fused with GST protein to produce GST-fusion proteins. The slides are simultaneously printed with polyclonal GST antibody to capture the newly synthesized GST fusion-proteins following coupled cell-free transcription-translation on the surface of the microarray. In both cases, newly synthesized proteins are captured through protein-protein (antigen-antibody) interactions. Although both technologies have been successfully employed, they also have their limitations. First, both platforms require a second protein, the antibody, to capture the synthesized fusion-protein. This antibody needs to be purified which adds to both labor and cost. Second, given that proteins (i.e. the capture antibody) need to be arrayed with the expression construct, maintaining the stability and integrity of the microarrays for extended periods of time remains an issue. We have addressed both of these issues by eliminating the need for antibody or other capture reagent to immobilize the newly synthesized proteins onto the microarray surface. In our system, the expression vector DNA not only directs the synthesis of each protein, but also serves to capture the protein at it's designated location on the microarray surface. Since only plasmid DNA is printed, array fabrication is simple and array stability is not an issue. To accomplish this we have exploited the high-affinity binding (∼3–7×10 ^−13^ M) of *E. coli* Tus protein to Ter, a 20 bp DNA sequence involved in the regulation of *E. coli* DNA replication [12], [13]. In our system, each protein of interest is synthesized as a Tus fusion protein and each expression construct directing the protein synthesis contains embedded Ter DNA sequence. The embedded Ter sequence functions as a capture reagent for the newly synthesized Tus fusion protein.

## Methods

### A. Construction of base microarray plasmid

For convenience, a recombinational cloning system was used (Invitrogen, Carlsbad, CA). First, a destination vector was made using Tus as the carboxy fusion partner. A modified, Tus (E47Q) with higher affinity for the Ter DNA sequence [12] was amplified from plasmid DNA by standard procedure. Oligos used for Tus amplifications were:

Forward- 5′-ATT TTA **GCT AGC** GGA GGT GCG CGT TAC GAT CTC GTA GAC CGA CTC-3′ and Reverse 5′-TATATT **CAA TTG** TTA atg atg gtg atg atg gtg ATC TGC AAC ATA CAG GTG CAG CCG TGG 3′.

Restriction sites NheI and MunI are indicated as bold and underlined. A six-histidine tag (small letters) was incorporated in reverse oligo so that Tus will be his-tagged for downstream identification.

The PCR product was purified, digested with NheI and MunI, run on an agarose gel, and the fragment excised. The fragment was then cloned into a derivative pDest47 (Invitrogen, Carlsbad, CA) termed pDest472 that had been digested with the same enzymes to create pDest 472-Tus. Correct clones were selected by digestion and verified by sequencing.

A Ter site (bold) was synthesized by annealing two complementary oligos:

CCGGC **CACTTTAGTTACAACATACTTATT**AT

CGA**TAATAAGTATGTTGTAACTAAAGTG**G

Following annealing, these oligos form a double stranded Ter site with ClaI and NgoMIV overhangs. The annealed oligo was cloned in pDest 472-Tus digested with NgoMIV and ClaI to create pDest Microarray TT-1. The clone was verified by sequencing. This is the base plasmid to clone any protein of interest by recombinational cloning.

In addition to a wild type Ter site, a mutant Ter site was also tested for Tus fusion capture. The mutant Ter site was obtained during the course of cloning the wild-type Ter site. The sequence of the mutant was found to be:

**CACTTTAGTTACAACATATTTATT**

The site of the mutation is underlined. It has been shown that mutation at this particular site will reduce binding affinity by almost 4-fold [11]. This position is equivalent to position 6 according to the nomenclature in the manuscript. The Ter sequence has been presented in reverse orientation [11].

### B. Construction of GFP fusion plasmid

As a proof-of-concept, we cloned GFP as a fusion with Tus. pEL100 contains eGFP gene in pDonr223 (Invitrogen, Carlsbad, CA). It contains a Kozak sequence upstream of ATG and no stop codon at the C-terminus. Thus, upon recombinational cloning into pDest Microarray TT-1, GFP will be fused in frame with Tus. Recombinational cloning was performed as per manufacturer's (Invitrogen, Carlsbad, CA) directions. Finally, the clone was sequenced to confirm correct insertion.

### C. Microarray Fabrication

Microarray protein expression vectors were prepared in 3× standard saline citrate (SSC) in a 384-well plate (Genetix, Boston, MA) and arrayed on nitrocellulose coated “Fast Slides” (Schleicher & Schuell BioScience, Keene, NH) using a Microgrid II microarray robot at 50% humidity. Microarray features were printed at a spacing of 0.55mm (center to center) with 1.2mm spacing between each sub array. After printing, microarrays were baked at 80°C for 30 min. Slides were blocked with 0.1% PVP/0.05% Tween 20 for 1 h prior to expression.

To confirm uniform DNA spotting, a sample slide from each print set was stained for DNA content using the IDT (Coralville, IA) Cy3-SpotQC detector oligo (9mers) diluted in 0.1% PVP/0.05% Tween 20 buffer. Following incubation, slides were washed 1× for 3 minutes in 10×SSC, 0.2% Sarkosyl, followed by a second wash in 10×SSC for one minute. After a third wash in 2×SSC, slides were dried and scanned with an Axon GenePix 4000 scanner (Figure S1).

### D. *In situ* Expression of Proteins

*In situ* expression was performed using a cell-free expression system (TNT Quick coupled transcription/translation system (Promega, Madison, WI)). In brief, 30 µl of rabbit reticulocyte lysate, supplemented with methionine, was added directly to the slide and incubated in a water bath. Expression and immobilization were carried out at 30°C for 1.5 hours followed by a 2 hour incubation at 15°C.

### E. Confirmation of Expression and Immobilization of Expressed Proteins

Expression of GFP-Tus protein was confirmed with a Cy3–Cy5 labeled antibody to the poly-histidine (poly-his) tag. Prior to incubation with labeled antibody, slides were blocked for 1 hr with 0.1% PVP/0.05% Tween 20. Monoclonal antibodies to poly-his, GFP (Sigma-Aldrich, St Louis, MO), and beta-globin (Novus, Littleton, CO) were labeled with fluorescent dye N-hydroxysuccinimide (NHS) ester-linked Cyanine 3 (Cy3) and Cyanine 5 (Cy5) (Amersham, Piscataway, NJ). In brief, 90 µl of antibody diluted to the concentration of 0.55 mg/ml in 0.1 M sodium bicarbonate/carbonate buffer pH 9.0 was mixed with 20 µl of 60 µM of Cy3 or Cy5 in sodium bicarbonate/carbonate buffer and incubated on ice. After reaction had proceeded for 90 minutes, 8 µl of Blocking Buffer (BD Biosciences, San Jose, CA) was added to the solution to quench the reactions and the solutions were allowed to sit for another 30 min with additional mixing approximately every 10 min. Unbound dye was removed by passing each sample through a size-exclusion chromatography spin column (sephadex G-15 (Sigma Aldrich, St. Louis, MO). Molar concentration for labeled protein and dye were calculated. The Cy5- labeled anti-his was mixed with equal amount of the Cy3-labeled anti-his and diluted in microarray hybridization buffer (0.1% PVP/0.1% Tween 20). Hybridization to the array was performed in an incubation chamber at 4°C with gentle rocking for at least 12 h. After incubation, slides were washed 3× for 5 minutes each in 10× PBS/0.05% Tween 20, followed by one wash in 10× PBS for one minute. All washes were performed at 4°C. Slides were dried and scanned on an Axon GenePix 4000 scanner (Union City, CA), and fluorescence data were collected and evaluated with the GenePix Pro 5.0 software. For the microarray imaging, the Axon GenePix 4000 scanner was set at 100% laser power and 350 PMT gain.

### System Design

a) Plasmid DNA encoding protein of interest (POI) was constructed such that the protein of interest (POI) is fused with an *E. coli* protein called Tus. b) The plasmid also contains one or more Tus binding sites termed Ter. Tus protein binds the Ter DNA sequence as a monomer with very high affinity, ∼3–7×10 ^−13^ M [11]. c) Plasmids are arrayed by a commercially available microarray printing robot. d) The POI-Tus fusion protein is synthesized on the microarray by coupled cell-free protein synthesis (either mammalian or prokaryotic). If the cocktail is derived from *E. coli*, it is made from a Tus minus strain. e) Affinity of the expressed POI-Tus fusion protein for the Ter sequence is significantly greater than the antigen-antibody affinity described by either Nord et al.[9] or Ramachandran et al [10].

## Results

**Figure 1:**
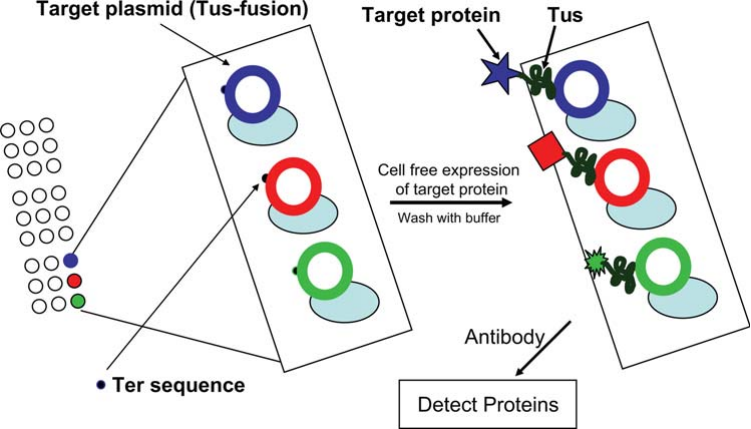
Concept of protein microarray on demand.

The design of the expression construct (pDest-Microarray TT-1) and basic concept of the array platform are shown in Figure 1 and Figure S1. The basic expression vector is based on the Gateway (Invitrogen, Carlsbad, CA) recombinational cloning system making it easier to generate libraries of constructs. To validate the Tus-Ter DNA-binding protein system for the development of an *in situ* self assembling protein array, as well as demonstrate the specificity of Tus-Ter binding, we printed a microarray containing a set of clones in pDest-Microarray TT-1 encoding a GFP-Tus-His6 fusion-protein and a Ter site, an identical vector with a point mutation in the Ter site, and a third construct without a Ter site.

**Figure 2:**
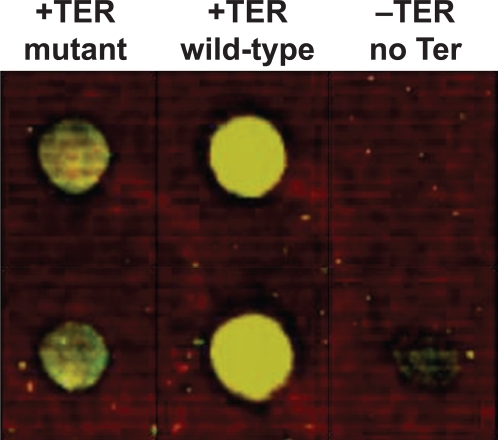
Exploiting the specificity of Tus:Ter interaction. Plasmid vectors encoding a green florescent protein (GFP)- TUS - poly-histidine fusion protein and a Ter sequence containing a point mutation (pMUT), a wild-type Ter sequence (pNOMut), and no Ter sequence were immobilized on the surface of a microarray, incubated in a cell-free rabbit reticulocyte transcription/translation extract, and hybridized with Cy-labeled anti-histidine antibody.

Anti-his antibody was labeled with Cy3 and Cy5, mixed in equimolar amounts, and hybridized to this microarray. The results are shown in Figure 2. We observed significant signal intensities (arbitrary units of 14000 and 20574 from Cy5 and Cy3 respectively) corresponding to the vector containing a wild-type Ter site (Fig. 2), confirming the expression and binding of the GFP-Tus fusion protein. In contrast for the vector without any Ter site, no significant signal was observed (arbitrary units of 0 and 2463 from Cy5 and Cy3 respectively; Fig. 2). The no-Ter/wild-type Ter signal ratio (−TER/+TER) is 0 for Cy5 and 0.12 for Cy3, consistent with significantly higher binding of the fusion protein to plasmid containing wild-type Ter as compared to plasmid without any Ter. Similarly, the vector containing a point mutation in the Ter site showed low to moderate signal (arbitrary units of 4738 and 10920 from Cy5 and Cy3 respectively; Fig. 2). The mutated-Ter/wild-type Ter signal ratio (mTER/+TER) is 0.34 for Cy5 and 0.53 for Cy3, indicating that a mutation in the Ter site results in reduced binding efficiency to the fusion protein. These data are in complete agreement with previous reports that Tus binds to the same mutated Ter with 4–6 fold lower efficiency [Bibr pone.0003265-Henderson1] [12. 13] as well as our own calculations of Tus: Ter and Tus: mutant Ter binding efficiencies and off rates (Table S1). These data demonstrate that an intact Ter sequence is necessary and sufficient for optimal binding to the Tus protein, allowing the effective binding of Tus-fusion proteins to Ter site(s) present in the vector and supporting the application of the Tus-Ter system for protein microarray fabrication.

In a parallel set of experiments, we have extended these findings to another DNA-binding protein system (lacI/LacO) and demonstrate that they can also function to mediate protein microarray fabrication in a similar manner (DC, CB, KS, JH, and DM; data not shown).

**Figure 3:**
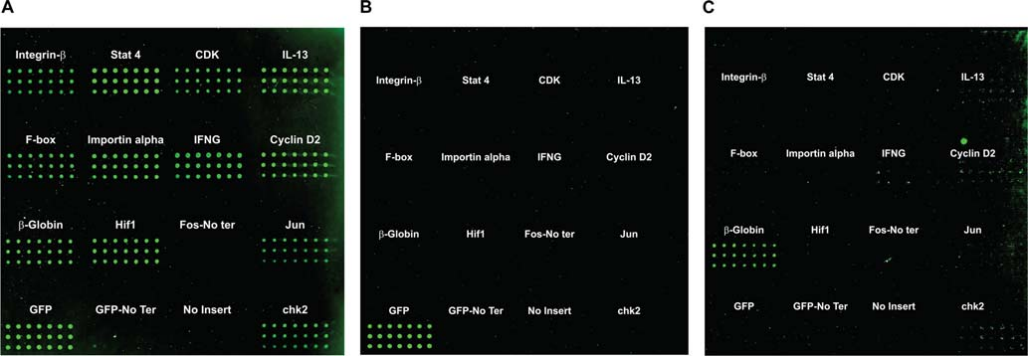
Microarray feature expression and capture specificity. pNOMut variants encoding different proteins fused to TUS - poly-histidine were immobilized on the surface of a microarray and incubated in a cell-free rabbit reticulocyte transcription/translation extract. Duplicate microarrays were then hybridized with cy-labeled monoclonal antibodies specific for A) His, B) GFP, and C) human *B*-globin.

As a more direct test of this platform, expression plasmids encoding 14 different proteins were immobilized onto the surface of a microarray. Expression from each of the constructs was confirmed by probing with a labeled antibody directed against the His-tag engineered into each construct (Fig. 3A). As expected, although an equal amount of DNA was printed for each feature, the relative amount of protein produced and retained by each construct varied modestly, presumably due to characteristic differential transcription/translation efficiencies (Fig. 3A and Figure S2). To validate that the individual targeted proteins were indeed expressed and captured at their designated location on the microarray, replicate arrays were probed with antibodies directed against the unique fusion partners specific for each construct (Fig. 3B and 3C). As shown, each of the target proteins was expressed and captured at a specific and designated location that was pre-determined by the insert encoded in the expression construct printed.

## Discussion

We have developed a simple and cost effective strategy for the rapid generation of protein microarrays. Because only DNA expression constructs are printed, the inherent cost, stability, and technical limitations most commonly associated with other protein microarray strategies are eliminated. As, shown in Figure 1 and Figure S1, the microarray is fabricated by the printing of DNA expression constructs that function to not only direct the synthesis of the desired protein, but also as the ‘capture reagent’ for immobilization of the encoded protein onto the microarray surface. The ‘capture reagent’ function of the printed DNA expression constructs is mediated by the specific and high-affinity binding (∼3–7×10 ^−13^ M) of *E. coli* Tus protein to Ter, a 20 bp DNA sequence. In Table S1 we show that both the specificity and the high-affinity binding, characteristic of wild-type Tus protein for Ter, is also true for the cloned versions of Tus:Ter interaction is maintained in a coupled in vitro transcription/translation system within a microarray environment. Finally, in Figure 3 we demonstrate that not only are the designated proteins specifically expressed, but that the individual expression constructs encoding each individual protein exclusively captures it's encoded protein without detectable diffusion or ‘bleeding’ to adjacent features on the microarray.

These data support the utility and effectiveness of this platform as a method for the high-throughput production of low-cost protein microarrays for the study of protein-protein, protein-nucleic acid, or protein-small molecule interaction. The open format of these arrays, together with their long ‘shelf-life’ and simple low-cost printing scheme, make this a cost-effective, versatile, production friendly platform amendable to a wide variety of uses and applications.

## Supporting Information

Figure S1 Design of expression construct and basic microarray fabrication schema
(0.67 MB TIF)

Figure S2 Validation of microarray printing. Different proteins fused to TUS - poly-histidine were immobilized on the surface of a microarray and stained for DNA content as described in Materials and Methods.
(9.91 MB TIF)

Table S1 (0.03 MB DOC)
