# Supplementary figures and images for: Protein Microarray On-Demand: A Novel Protein Microarray System
Source: PLoS One. 2008 Sep 24;3(9):e3265. doi: 10.1371/journal.pone.0003265 (PMC2533396; doi:10.1371/journal.pone.0003265)

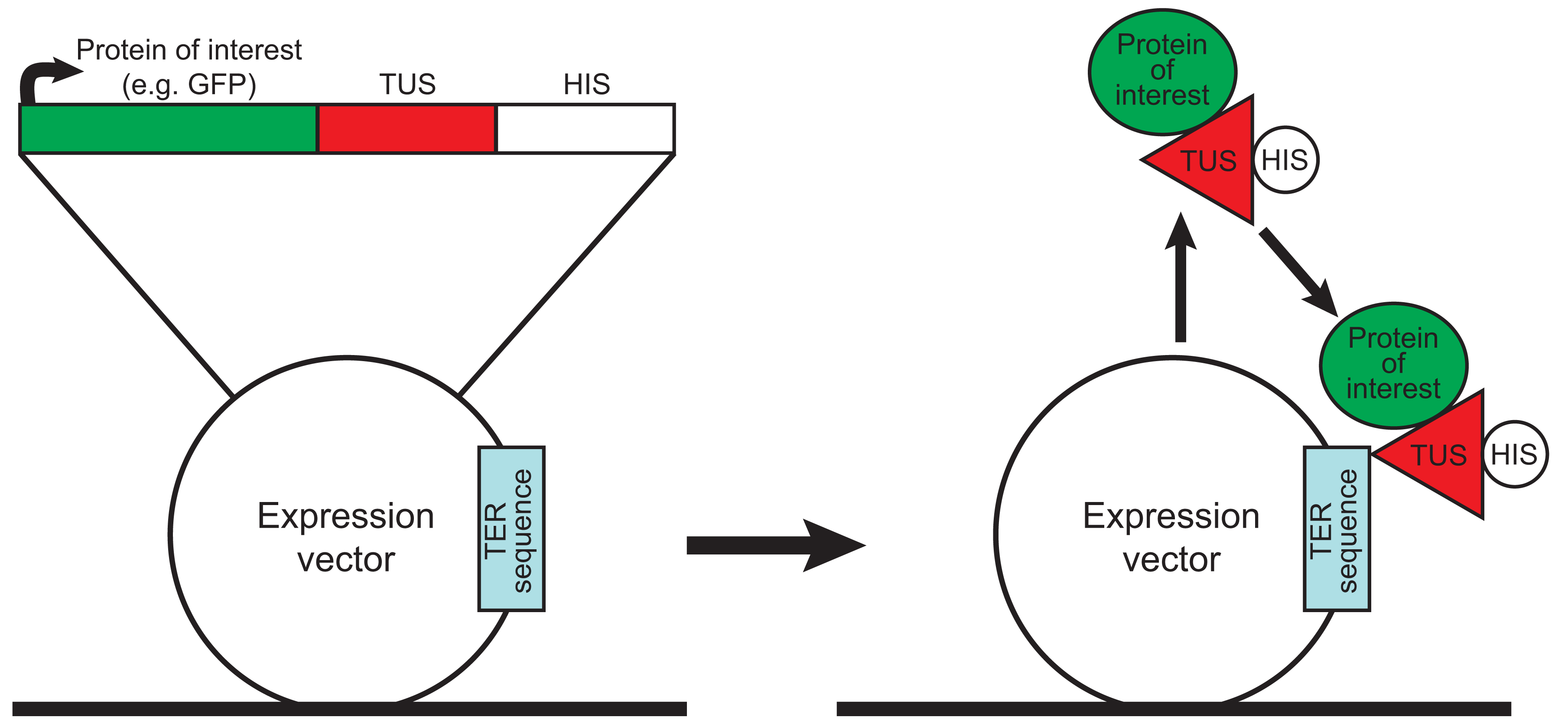

Supplement: Figure S1 — Design of expression construct and basic microarray fabrication schema (0.67 MB TIF) [file pone.0003265.s001.tif]

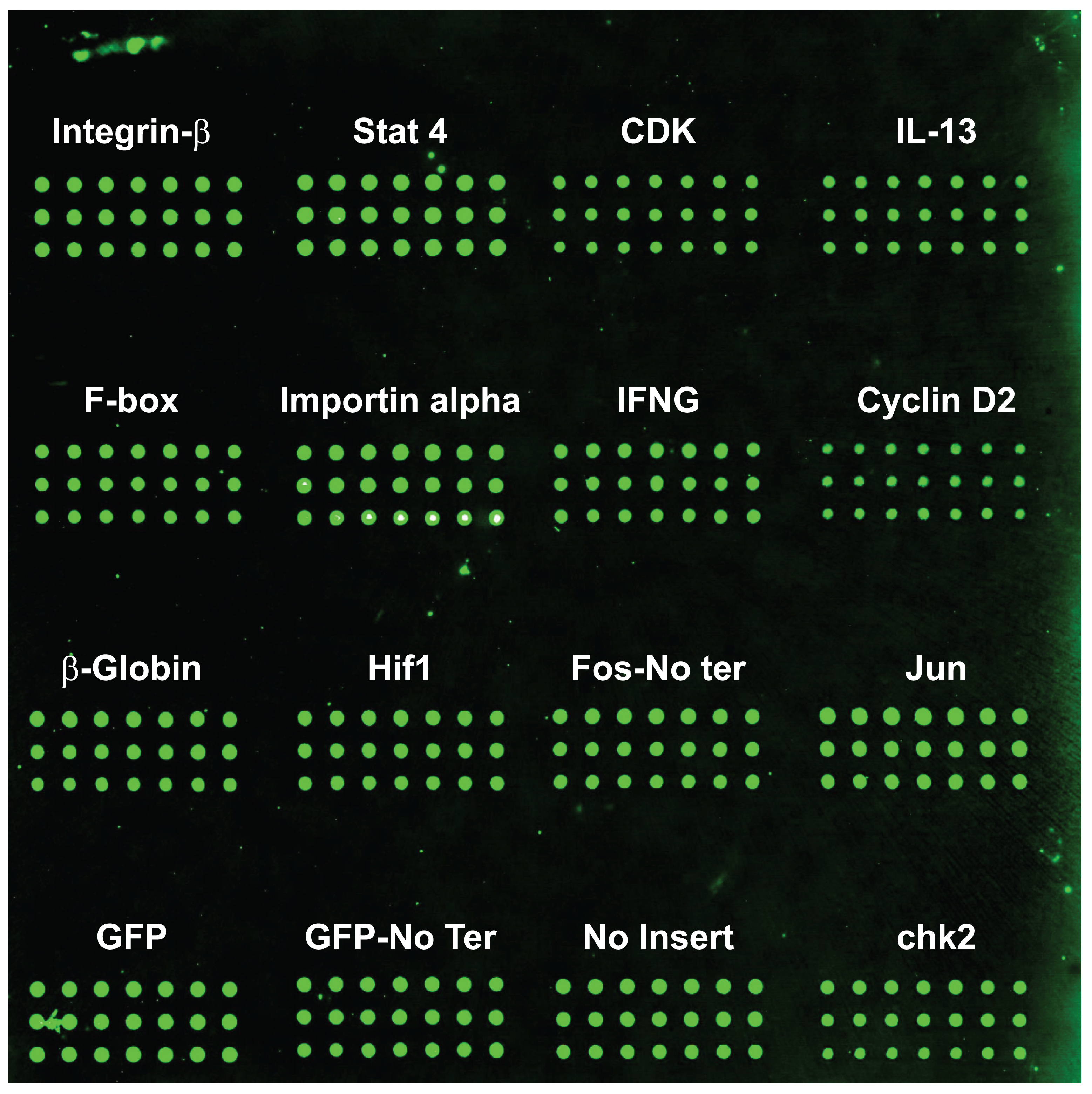

Supplement: Figure S2 — Validation of microarray printing. Different proteins fused to TUS - poly-histidine were immobilized on the surface of a microarray and stained for DNA content as described in Materials and Methods. (9.91 MB TIF) [file pone.0003265.s002.tif]
