# Supplementary material for: Protein Microarray On-Demand: A Novel Protein Microarray System
Source: PLoS One. 2008 Sep 24;3(9):e3265. doi: 10.1371/journal.pone.0003265 (PMC2533396; doi:10.1371/journal.pone.0003265)
Supplement: Table S1 — (0.03 MB DOC) [file pone.0003265.s003.doc]

Table S1. **High Affinity binding of Tus protein to the Ter DNA sequence.**

**Protein** **Ter affinity (M)** **T ½ (minutes)**

Wild-type Tus 7-8 X 10^-13^ 160

Tus clone***** 2 X 10^-13^ 350

GFP-Tus clone***** fusion 3-5 X 10^-12^ 800

*****Tus clone E47Q described in J. Biol. Chem **272**, 26448-26456 (1997)

Tus: Ter binding affinities and ‘off-rates’ were calculated as described in Materials and Methods.
